# Pharmacokinetic, metabolic stability, plasma protein binding and CYP450s inhibition/induction assessment studies of N-(2-pyridylmethyl)-2-hydroxiymethyl-1-pyrrolidinyl-4-(3-chloro-4-methoxy-benzylamino)-5-pyrimidine-carboxamide as potential type 5 phosphodiesterase inhibitors

**DOI:** 10.1080/19768354.2019.1614091

**Published:** 2019-05-16

**Authors:** Haijun Qu, Xiaoxiao Hu, Xiaoli Shi, Chuan Wang, Longyuan Wang, Guoping Wang

**Affiliations:** Department of Pharmacy, Affiliated Hospital of Qingdao University, Qingdao, People’s Republic of China

**Keywords:** Plasma protein binding, metabolic stability, cytochrome p450 inhibition and induction, pharmacokinetics, type 5 phosphodiesterase inhibitors

## Abstract

N-(2-pyridylmethyl)-2-hydroxiymethyl-1-pyrrolidinyl-4-(3-chloro-4-methoxy-benzylamino)-5-pyrimidine-carboxamide (NHPPC) is a new potential of type 5 phosphodiesterase (PDE5) inhibitors, synthesized from the avanafil analogue for the treatment of erectile dysfunction. The targets of this article were to assess plasma protein binding, liver microsomal metabolic stability, inhibition and induction on cytochrome P450 isozymes and the pharmacokinetics of NHPPC. Equilibrium dialysis technique was applied to determine Plasma protein binding (PPB) and NHPPC was evaluated in male Sprague–Dawley rats and Beagle dogs in vivo pharmacokinetic. The NHPPC was highly bound to plasma proteins in rats, dogs and human tested and the mean values for PPB rate were 96.2%, 99.6% and 99.4%, respectively. After in vitro liver microsomes incubated for 60 min, the percent remaining of NHPPC was 42.8%, 0.8% and 42.0% in rats, dogs and human, respectively. In vitro intrinsic clearance was found to be 0.0233, 0.1204 and 0.0214 mL/min/mg protein in rat, dog and human liver microsomes of NHPPC, respectively. NHPPC showed no significant inhibitory effects on major CYP450 enzymes, and had no significant induction potential on CYP1A2 and CYP3A4. Following oral administration in rats and dogs, *t*_max_ was 6 and 0.5 h, respectively. The clearance for NHPPC was 1.19 and 1.46 L/h/kg in rats and dogs, respectively. And absolute bioavailability in rat and dog were approximately 34.5% and 53.1%, respectively. These results showed that NHPPC has a good development prospect.

## 1. Introduction

**Figure 1.**
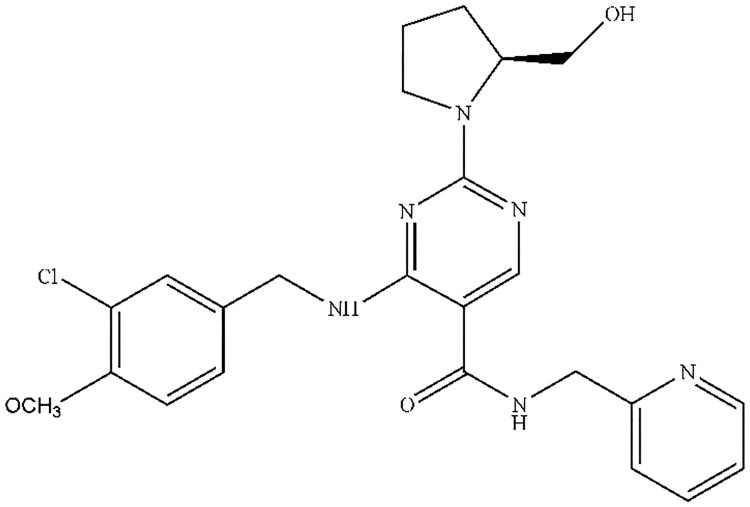
Chemical structure of NHPCC.#

N-(2-pyridylmethyl)-2-hydroxiymethyl-1-pyrrolidinyl-4-(3-chloro-4-methoxy-benzylamino)-5-pyrimidine-carboxamide (NHPPC, Figure 1), a potential of type 5 phosphodiesterase inhibitors, was avanafil modified structural analog. Avanafil is a type 5 phosphodiesterase (PDE5) inhibitor approved for erectile dysfunction (ED) by the FDA on 27 April 2012. According to relevant literature (Zhao et al. 2012; Belkoff et al. 2013), avanafil is effective and safe for ED. NHPPC may be effective in treating ED and reduce side effects through further optimization. The preliminary efficacy experiment of rabbit model in vivo showed that NHPPC has stronger than avanafil. This data cannot be published publicly for the time being, and an article will be written in the future. It is noteworthy that ED is widespread complaint, affecting 150 million men worldwide (Selvin et al. 2007). PDE5 inhibitors including avanafil, tadalafil, vardenafil and sildenafil have been widely accepted as first-line therapy for ED (Doggrell 2005; Yuan et al. 2013). But these conventional therapeutic drugs have not yet met with therapeutic purposes, and there are some adverse reactions. Therefore, the novel highly selective PDE5 inhibitors with strong efficacy and fast onset of action have great significance and economic value, and improve the safe use of drugs to prevent adverse reactions. Compared with other similar drugs, avanafil provide a rapid onset of action with long-term efficacy in treating ED (Limin et al. 2010; Belkoff et al. 2013). But the most common adverse reactions reported in drug’s instructions are headache, dizziness, cold symptoms, flushing, nasal congestion, back pain and visual impairment. It can also have more severe side effects such as: change or loss of vision and/or hearing, priapism, chest or stomach pain. Ananafil as new high selectivity PDE5 inhibitors, analogs of its structure will be potential biological activity of drug molecules. It may be that we need to discover and develop new compounds, which can improve the safe use of drugs to prevent adverse reactions. So in the early research stage, we need to conduct a series of in vivo and in vitro studies including plasma protein binding (PPB) (Clark and Grootenhuis 2002; Vande and Gifford 2003), metabolic stability (Xu et al. 2002; Baranczewski et al. 2006; Zhang et al. 2007)and a simple pharmacokinetic (PK) model.

## 2. Materials and methods

### 2.1. Chemicals, reagents and instruments

N-(2-pyridylmethyl)-2-amino-4-(3-chloro-4-methoxy-benzylamino)-5-pyrimidine-carboxamide (NHPPC) and the internal standard (IS) with purities of >95%, vardenafil, were obtained from MedChemExpress (Shanghai, China). The positive control drug (propafenone and warf) were obtained from Sigma Chemical Co. (St. Louis, MO, USA). Pharmaceutical grade dimethyl sulfoxide (DMSO, purity >99.98%) was supplied by Zhonglan Industry Co., Ltd. (Shanghai, China). Pharmaceutical grade Hydroxypropyl-beta-cyclodextrin (HP-β-CD, purity >99.9%) was purchased from Shandong Binzhou Zhiyuan Biotechnology Co. Ltd. (Shangdong, China). Control human, Sprague–Dawley rat and Beagle dog plasma (EDTA-K2 anti-coagulant) was purchased from Bioreclamation (Hicksville, NY, USA) for plasma protein binding experiments. Potassium dihydrogen phosphate, dipotassium hydrogen phosphate and sodium hydroxide were obtained from Sinopharm Chemical Reagent Co., Ltd. (Shanghai, China). Equilibrium dialysis membranes with a molecular mass cut-off of 0.8–14 kDa were purchased from Viskase (Darien, USA). HPLC-grade formic acid (FA), DMSO, isocitric acid (IA), magnesium dichloride (MgCl_2_), β-Nicotinamide adenine dinucleotide phosphate (NADP) and isocitric dehydrogenase (IDH), henacetin, diclofenac, S-mephenytoin, dextromethorphan, Midazolam, testosterone, α-Naphtoflavone, sulfaphenazole, N-3-benzylnirvanol, quinidine, ketoconazole, omeprazole and rifampin were purchased from Sigma-Aldrich (Steinheim, Germany). Pooled human, rat, and dog liver microsomes were purchased from BD Gentest (Woburn, MA, USA). Collagenated 48-well plate was obtained by BD Biocoat. HPLC-grade acetonitrile and methanol were purchased from Merck (Darmstadt, Germany). Deionized water was obtained from a Milli-Q plus water purification system (Milli-Q, Millipore Corp., MA, USA). All other chemicals and reagents used were of either HPLC or analytical grade.

All LC analysis was carried out on a liquid chromatographic system (Kyoto, Japan) consisting of Shimadzu model LC-20A pump, an autosampler (SIL-20ACHT, UFLC), a column oven (CTO-20A), and an on-line degasser (DGU-20A3). The autosampler temperature was maintained at 4 °C.

### 2.2. Animals and dose formulation

Sprague–Dawley (SD) rats (180–220 g, 8 weeks) were purchased from Beijing Vital River Laboratory Animal Technology Co., Ltd. (Beijing, China), and Beagle dogs (age: 8 months, body weight: 6-9 kg) were purchased from Beijing Marshall Biotechnology Co., Ltd. The room was controlled and monitored for humidity (40%–70%) and temperature (20 °C–25 °C) with 10–20 air changes/h, and a 12-h light/dark cycle. All animals were fasted for 12 h before experiment with water ad libitum and the feed was provided 4 h post-dose. The food and water were certified to adhere to the national standards GB14924.3–2010 and GB14925–2010. For preparation of standards and quality controls, blank plasma was collected from adult healthy SD rats and Beagle dogs. All animal procedures were conducted according to the Institutional Animal Care and Use Committee (IACUC) for animal experimentation, and the protocol was approved by the Animal Ethics Committee of Qingdao University.

Solution for the intravenous (IV) and oral (PO) dosing formulation were prepared freshly before dosing as follows: appropriate amount of NHPPC was weighted accurately and dissolved in appropriate volume of DMSO, and followed by adding appropriate volume of 6%HP-β-CD in aqueous solution while vortexing. Then the resulting solution, containing 2% DMSO and 98% 6%HP-β-CD in aqueous solution, was filtered by 0.22 µm membrane. The final concentration of NHPPC was 1.0 mg/mL. The IV dose volume was 1 mL/kg for rats and dogs, and the PO dose volume was 2 mL/kg for rats and 1 mL/kg for dogs.

### 2.3. Preparation of working solutions

The NHPPC and vardenafil (internal standard, IS) stock solutions of 1 mg/mL were prepared in an appropriate volume of DMSO for standard and quality control samples. The NHPPC and the positive control drug (propafenone and warf) stock solutions of 10 mM were prepared in an appropriate volume of DMSO for plasma protein binding and metabolic stability studies. The working solutions of NHPPC were obtained by serial dilution using acetonitrile: H_2_O (1:1, v/v) mixture as a diluent. The final concentrations of NHPPC working solution was 20, 50, 100, 250, 500, 1000, 2000, 5000, 10,000, and 20,000 ng/mL for standard calibration, and 40, 2000, and 16,000 ng/mL for quality control samples. The IS stock solution was diluted to 100 ng/mL by step-wise dilution of the stock solution with acetonitrile.

### 2.4 Preparation of standard calibration and quality control samples

The calibration curve standard samples were prepared by spiking 450 μL of blank rat or dog plasma with 50 μL of each NHPPC working solution to yield. The final concentrations for the NHPPC standard samples were 2, 5, 10, 25, 50, 100, 250, 500, 1000 and 2000 ng/mL. Quality control (QC) samples for NHPPC were prepared at three different concentrations (4, 200 and 1600 ng/mL) in the same way as the plasma samples for calibration. Stock solutions were stored at –4°C until analysis.

### 2.5. Preparation of samples

The samples including plasma of calibrators and QCs, plasma protein binding, metabolic stability study and unknown samples of PK studies were processed by simple protein precipitation with acetonitrile. The unknown samples were thawed at room temperature. A 50 μL aliquot from each sample was pipetted into centrifuge tubes, followed by adding 200 μL of the IS working solution and vortexed for 5 min. The mixture was centrifuged at 12,000 rpm for 5 min at 4°C. A 50 aliquot of each separated supernatant was transferred into a clean 96-well plate which was contained 150 μL of water. Then an aliquot of 10 μL of each samples was vortexed for 5 min and injected onto LC-MS/MS for analysis.

### 2.6. LC-MS/MS conditions

Analytes and IS peaks were carried out on an Agilent XDB C_18_ column (2.1 × 50 mm, i.d.; 5 μm, Agilent, CA, USA) The mobile phase consisted of (A) 0.1% FA in water and (B) 0.1% FA in acetonitrile at a flow rate 0.5 mL/min. The gradient elution program was as follows: 5% B as initial mobile phase, 0–0.3 min, 0.3–0.8 min, linear gradient from 5% to 95% B; 0.8–2.0 min, isocratic elution with 95% B; 2.0–2.3 min, linear gradient from 95% to 5% B, 2.3–3.0 min, isocratic elution with 5% B. The column temperature was maintained at 35 °C and the injection volume at 10 μL. Mass spectrometric detection was performed on API4000 Qtrap mass spectrometer with an electrospray ionization (ESI) interface operating in positive ion mode, was manufactured by Applied Biosystems (Toronto, Canada). The MS/MS system was operated at unit resolution in the multiple reaction monitoring (MRM) mode, and the monitored transitions were *m*/*z* 483.1→375.2 for NHPPC, *m*/*z* 489.4→151.3 for IS, *m*/*z* 309.0→163.1 for warf and *m*/*z* 342.4→116.2 for propafenone, respectively. The mass spectrometry conditions were as follows: CAD: medium, CUR: 20 psi, Gas1: 55 psi, Gas2: 55 psi, ISV: 5000 V, TEM: 600°C, EP: 10 V, CXP: 12, and dwell time: 100 ms. Compound parameters like DP and CE were optimized at 100 and 40 V for NHPPC, 120 and 60 V for IS, 48 and 15 V for warf and 100 and 50 V for propafenone, respectively. Analyst 1.6.1 from AB SCIEX was used for outputting raw chromatograms and generating concentration results.

### 2.7. Plasma protein binding (PPB) study

The phosphate buffer (100 mM) and sodium hydroxide solution (1.0 M) were prepared in deionized water. The stability in plasma was evaluated at 0.2 and 1.0 μM of NHPPC and the positive control warf for each species by incubating at 37°C for 6 h before proceeding with PPB study. Non-specific binding potential of NHPPC and warf to dialysis membrane was assessed by spiking them into phosphate buffer and incubating for 6 h. NHPPC and warf were stable in plasma for 6 h and showed no non-specific binding to dialysis membrane. PPB was determined by equilibrium dialysis method. An aliquot (150 μL) of NHPPC spiked plasma and phosphate buffer (pH 7.4) was added into the donor side and the receiver side of each designated well. The plate was covered with adhesive sealing film to prevent evaporation and placed in a water-bath shaker kept at 37°C for 6 h with a rate of 50 rpm. The cross-species assessment of bound fraction was conducted with 6 h equilibration in rat, dog, and human plasma at NHPPC and warf concentrations of 0.2 and 1.0 μM, respectively. At the end of 6 h, aliquots were taken from each side of each well (*n* = 3) and kept in a −20°C freezer prior to analysis. The stability, device recovery and PPB were calculated using the following equations:

$$Remaining\;at\;6\;h\;(\text{\%})=\frac{C_{6h}}{C_{0h}}\times 100\text{\%},$$

Device recovery (%)=CDonor+CReceiverC6h×100%,

PPB (%)=CDonor−CReceiverCDonor×100%.

### 2.8 In vitro metabolic stability study

The phosphate buffer (pH 7.4, 100 mM) and (300 mM) were prepared in deionized water. NADP and IA were dissolved in phosphate buffer and MgCl_2_ solution and then added 20 μL IDH (18 units/mg protein) and mixed. NHPPC (1 mM) and positive control drug were prepared by dilution using DMSO and then the NHPPC and positive control drug working solution (50 μM) were obtained by dilution using methanol: H_2_O (1:1, v/v) mixture. Pooled human, rat and dog liver microsomes were added to phosphate buffer pH7.4 to a concentration 20 mg/mL. NHPPC was incubated with liver microsomes. The incubation solution of the final reaction conditions contained 50 mM potassium phosphate buffer (pH 7.4), 0.5 mg protein/mL microsomes, 3 mM MgCl_2_, 2 mM NADPH and 1 μM NHPPC or positive control drug in a final volume of 100 μL. In sample and positive control groups, NADPH was added into the incubation solutions. In the negative control group, water (the replacement of NADPH) was added into the incubation solutions. Samples were incubated aerobically in a shaking water bath at 37°C and the reaction was terminated by adding 300 μL of ice cold acetonitrile (contain 100 ng/mL IS) at different interval 0, 5, 10, 20, 30 and 60 min. While positive control and negative control were incubated aerobically in a shaking water bath at 37°C for 0 and 60 min, respectively. NHPPC and control concentrations were determined by LC-MS/MS at each time point (*n* = 3). Calculation of *t*_1/2_ and CL_int_ was performed by using following equations, where K is the elimination rate constant (min^−1^) and *Q_H_* is hepatic blood flow (mL/min/kg).

$$\begin{array}{rl} t_{1/2}= & \frac{0.693}{K}\\ CL_{int,in\;vitro}= & V_{D}\times K\\ V_{D}= & \frac{Volume\;of\;reaction\;mixture(mL)}{mg\;of\;protein\;per\;reation}. \end{array}$$

Hepatic intrinsic clearance was estimated by using the scaling factors and calculated by using the following equation (Lave et al. 1997).

$$\begin{array}{rl} CL_{\mathrm{int},in\;vivo}= & CL_{\mathrm{int}}\times mg\;microsomal\;protein\;per\;liver\\ & \times g\;liver\;per\;kg\;body\;weight, \end{array}$$

$$C_{\mathrm{int},H}=\frac{CL_{\mathrm{int},in\;vivo}\times Q_{H}}{CL_{\mathrm{int},in\;vivo}+Q_{H}},E_{H}=\frac{CL_{\mathrm{int}},H}{Q_{H}}.$$

### 2.9 Inhibition and induction on CYP isozymes

The stock solutions of the probe substrates phenacetin (20.0 mM), diclofenac (20.0 mM), S-mephenytoin (30 mM), dextromethorphan (10.0 mM), midazolam (10.00 mM) and testosterone (50.0 mM) were prepared using methanol, while α-Naphtoflavone (3.0 mM), sulfaphenazole (3.0 mM), N-3-benzylnirvanol (3.0 mM), quinidine (3.0 mM), ketoconazole (3.0 mM) and NHPPC (10.0 mM) were prepared using DMSO, and diluted with phosphate buffer (PB, 100 mM, pH 7.4) before added into the incubation system. The organic solvents in the final incubate were no more than 0.20%. The incubation medium was 100 mM phosphate buffered saline, and the final incubation volume was 200 μL containing 25 pmol/mL human liver microsomes CYP enzyme, probe substrate, 1 mM NADPH and NHPPC at 3.00 μM. The reactions were initiated after 5 min pre-incubation at 37°C and were terminated by the addition of 400 μL ice-cold acetonitrile. All incubations were conducted in triplicate, and the samples were stored at −80°C until analysis. Calculation of IC50 was performed by using following equations, where C is concentration of test compounds.

$$IC50=\frac{C\times (100-\%inhibiton\;at\;C)}{\%inhibition},(suppose\;Hill\;slope\;=\;1)$$

The hepatocytes were seeded on a collagenated 48-well plate for 24 h in an incubator at 37°C (moisturized, with 5% CO_2_). The hepatocytes were then incubated with the test article thereafter. The stock solutions of omeprazole (100 mM), rifampin (20.0 mM), testosterone (200 mM), phenacetin (100 mM) and NHPPC (50.0 mM) were prepared in DMSO, and were diluted using the cell culture medium. The DMSO content in the final solutions was 0.10%, the medium containing 0.10% DMSO was used as the blank control. The cells were co-incubated with the positive control inducers or NHPPC for 72 h and during the incubation the medium was changed every day. After the incubation, the medium was discarded and replaced with 200 μL phenol red-free culture medium containing 100 μM phenacetin (CYP1A2 probe substrate) or 200 μM testosterone (CYP3A4 probe substrate). After the incubation in the presence of the probe substrates at 37°C for 1 h, the supernatants were collected and stored at −80°C until analysis. All incubations were also conducted in triplicate. The generated metabolites of the CYP1A2 and CYP3A4 probe substrates were analyzed by LC/MS/MS methods. The induction effects of the positive control inducers and NHPPC were assessed by the amount of metabolites formed in the induced and uninduced hepatocytes. The percent induction of positive control was calculated using the following equation.

$$Percent\;induction\;of\;positive\;control(\text{\%})=\frac{(A_{t}-A_{n})\times 100\text{\%}}{A_{p}-A_{n}},$$

where *A_t _*= test sample activity, *A_n_* = negative control activity and *A_p_**_ _*= positive control activity.

### 2.10. In vivo pharmacokinetic (PK) study

For the iv experiment, male SD rats (*n* = 3) were given a single dose of NHPPC (1 mg/kg) over 1 min by dorsal venous arch of foot. Approximately 200 µL blood samples were collected at 5, 15, 30 min and 1, 2, 4, 6, 8 and 24 h post-dose via tail vein into heparinized (coated with 0.1% heparin in saline) tubes. For the oral experiments, the male rats (*n* = 3) were given a single dose of 2 mg/kg. Then the blood samples were collected at 10, 30 min and 1, 2, 4, 6, 8 and 24 h post-dose. Plasma was obtained after centrifugation at 8000 rpm for 6 min (4°C) within 10 min of blood-drawing and stored at –70°C until they were analyzed.

For the iv experiment, male Beagle dogs (*n* = 3) were given a single dose of NHPPC (1 mg/kg) over 1 min by forelimb saphenous vein. Approximately 500 µL blood samples were collected at 5, 15, 30 min and 1, 2, 4, 6, 8 and 24 h post-dose via small saphenous vein into heparinized (coated with 0.1% heparin in saline) tubes. For the oral experiments, the male rats (*n* = 3) were given a single dose of 1 mg/kg. Then the blood samples were collected at 10, 30 min and 1, 2, 4, 6, 8 and 24 h post-dose. Plasma was obtained after centrifugation at 8000 rpm for 6 min (4°C) within 10 min of blood-drawing and stored at –70°C until they were analyzed. The bioavailability (BA %) was calculated using the formula below:

$$F\text{\%}=\frac{AUC_{inf}\;(PO)\times Dose\;(IV)}{AUC_{inf}\;(IV)\times Dose\;(PO)}\times 100.$$

### 2.11. Data analysis

Data analysis including analyte/IS was acquired using Analyst version 1.6.1 software (AB SCIEX). All pharmacokinetic parameters (for example: *K*_10_, AUC_0–*t*_, AUC_0-∞_, *C*_max_, *T*_max_, *t*_1/2_, CL, *V_ss_*, MRT_0–∞_, etc.) were analyzed based on a non-compartmental model and using Pharsight WinNonlin version 6.3 (St. Louis, USA). The area under the concentration time curve (AUC_0–*t*_ and AUC_0–∞_) was calculated by linear trapezoidal rule. The natural logarithms (Ln) of the fraction remaining against incubation times (min) was plotted and the first-order rate constant of turnover is determined by a linear regression model. The final results (for example: the mean, standard deviation, the remaining, device recovery, *k_e_*, bioavailability, etc.) were electronically exported into Excel 7.0 (Redmond, USA) for data processing and formatting.

## 3. Results

### 3.1 Plasma protein binding

NHPPC was highly bound (>95%) to rats, dogs and humans plasma proteins. The PPB rates of NHPPC in rats were assessed to be 95.7% and 96.7% at 0.2 and 1.0 µM, respectively. The PPB rates of NHPPC in dogs were assessed to be 99.2% and 99.9% at 0.2 and 1.0 µM, respectively. The PPB rates of NHPPC in humans were assessed to be 99.3% and 99.5% at 0.2 and 1.0 µM, respectively. The plasma stability tests of NHPPC at 0.2 and 1.0 µM, respectively, at 37°C showed that NHPPC was stable for 6 h in the rats (100.9%), dogs (100.0%) and human (100.7%) plasma, with ≥99% NHPPC remaining after the 6 h incubation. In addition, non-specific binding was not observed with membrane or equilibrium device. All data of PPB studies of NHPPC and the positive control warf were summarized in Tables 1 and 2.

**Table 1:** . Stability of NHPPC and warf in plasma after 6 h incubation at 37°C.

Concentration (µM)	Mean drug remaining % (*n* = 3)					
	NHPPC			Warf		
	Rat	Dog	Human	Rat	Dog	Human
0.2	100.3 ± 1.93	99.6 ± 0.53	99.8 ± 1.37	99.2 ± 0.93	99.9 ± 1.46	99.0 ± 0.50
1	101.5 ± 1.26	100.4 ± 0.98	101.7 ± 1.50	99.7 ± 1.09	100.2 ± 2.05	99.6 ± 0.87
Mean	100.9 ± 1.01	100.0 ± 1.24	100.7 ± 1.23	99.5 ± 0.82	100.1 ± 1.87	99.3 ± 1.08

**Table 2.** The PPB and recovery of NHPPC and warf in plasma determined using an equilibrium dialysis method.

Concentration (µM)	Mean PPB % (*n* = 2) or mean device recovery % (*n* = 3)					
	NHPPC			Warf		
	Rat	Dog	Human	Rat	Dog	Human
0.2	95.7 ± 0.26	99.2 ± 0.12	99.3 ± 0.20	94.8 ± 0.36	95.1 ± 0.20	99.2 ± 0.23
1	96.7 ± 0.21	99.9 ± 0.38	99.5 ± 0.16	95.4 ± 0.23	95.3 ± 0.13	99.8 ± 0.29
Mean PPB	96.2 ± 0.18	99.6 ± 0.26	99.4 ± 0.13	95.1 ± 0.18	95.2 ± 0.04	99.5 ± 0.19
Mean recovery	98.6 ± 0.13	95.7 ± 0.30	101.2 ± 0.02	107.9 ± 0.03	100.7 ± 0.49	107.0 ± 0.25

### 3.2 In vitro metabolic stability

**Figure 2.**
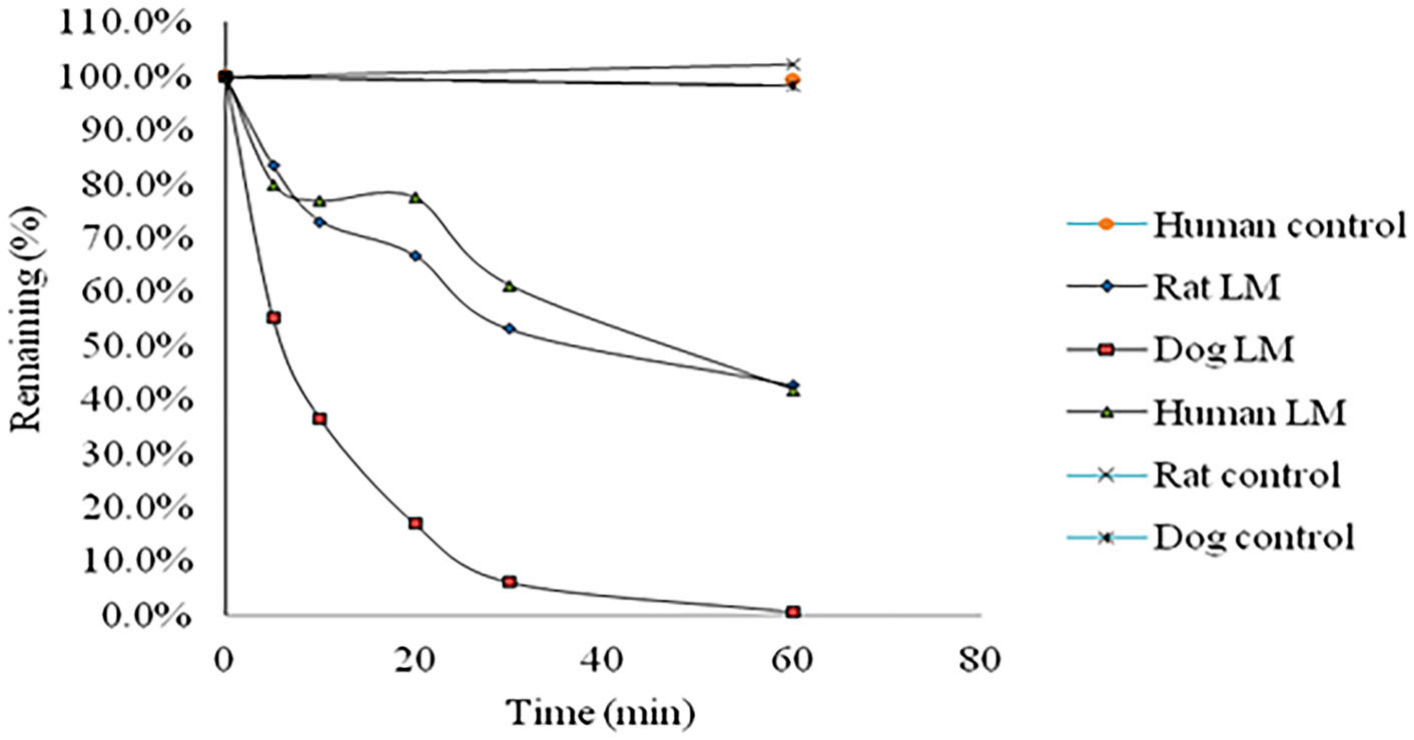
Time-dependent metabolic depletion profiles of NHPPC in rat, dog and human liver microsomes (LM) for: the reaction with NADPH and control without NADPH (% amount remaining vs. incubation time).

NHPPC incubated with microsomes without NADPH regenerating system for 60 min, and the percent remaining of NHPPC was 102.4%, 98.4% and 99.4% in rat, dog and human liver microsomes, respectively. After incubated for 60 min in the presence of NADPH, the percent remaining of NHPPC was 42.8%, 0.8% and 42.0% in rat, dog and human liver microsomes (Figure 2), respectively. After incubated for 60 min, the percent remaining of the positive control propafenone were <0.1%, 0.2% and 0.1% in rat, dog and human liver microsomes, respectively. The positive control was employed in order to ensure that microsomes and incubation conditions were appropriate to assess liver microsomal metabolic stability studies. The results indicated that the incubation system was normal. The determined parameters are *K_e_*, *t*_1/2_, CL_int, ivtro_, CL_H_ and regression co-efficient (*R*^2^) are shown in Table 3.

**Table 3.** Parameters of NHPPC in rat, dog and human liver microsomes.

Species	*K_e_* (min^−1^)	*t_1_*_/2_ (min)	*R*^2^	CL_int, ivtro_ (mL/min/mg)	CL_H_ (mL/min/kg)
Rat	0.0163	42.5	0.8434	0.0233	27.5
Dog	0.0843	8.2	0.9905	0.1204	25.3
Human	0.0150	46.2	0.9134	0.0214	9.80

### 3.3 Inhibition and induction

The changes of the human liver microsomes CYP isozyme activities after the co-incubation with NHPPC (3 μM) and the corresponding probe substrates were investigated and the IC50 values were calculated to assess the inhibition potential of NHPPC on CYP isozymes. The enzyme activities were reflected by the formed amount of the metabolites of the probe substrates. According to relevant literature (White 2000; Yan and Caldwell G 2001), the results showed that the IC50 values of NHPPC were greater than 100 μM for CYP1A2, CYP2C19, CYP2D6, CYP3A4 (midazolam based) and CYP3A4 (testosterone based), while 21.9 μM for CYP2C9. The results suggested NHPPC had no inhibition on CYP1A2, CYP2C9, CYP2C19, CYP2D6 and CYP3A4.

The changes of CYP1A2 and CYP3A4 activities in human primary hepatocytes of three batches after co-incubation with NHPPC at 0.10, 1.00 and 10.0 μM were investigated to evaluate the induction potential of NHPPC on CYP enzymes. Test compounds would be considered as inducers when the percent induction of positive control ≥40% (US FDA 2000). The results showed that the percent induction of NHPPC after the treatment at 0.10, 1.00 or 10.0 μM was less than 40% of that from the positive control for both CYP1A2 and CYP3A4; suggesting NHPPC had no induction on CYP1A2 and CYP3A4 in human primary hepatocytes.

### 3.4 In vivo pharmacokinetics

**Figure 3.**
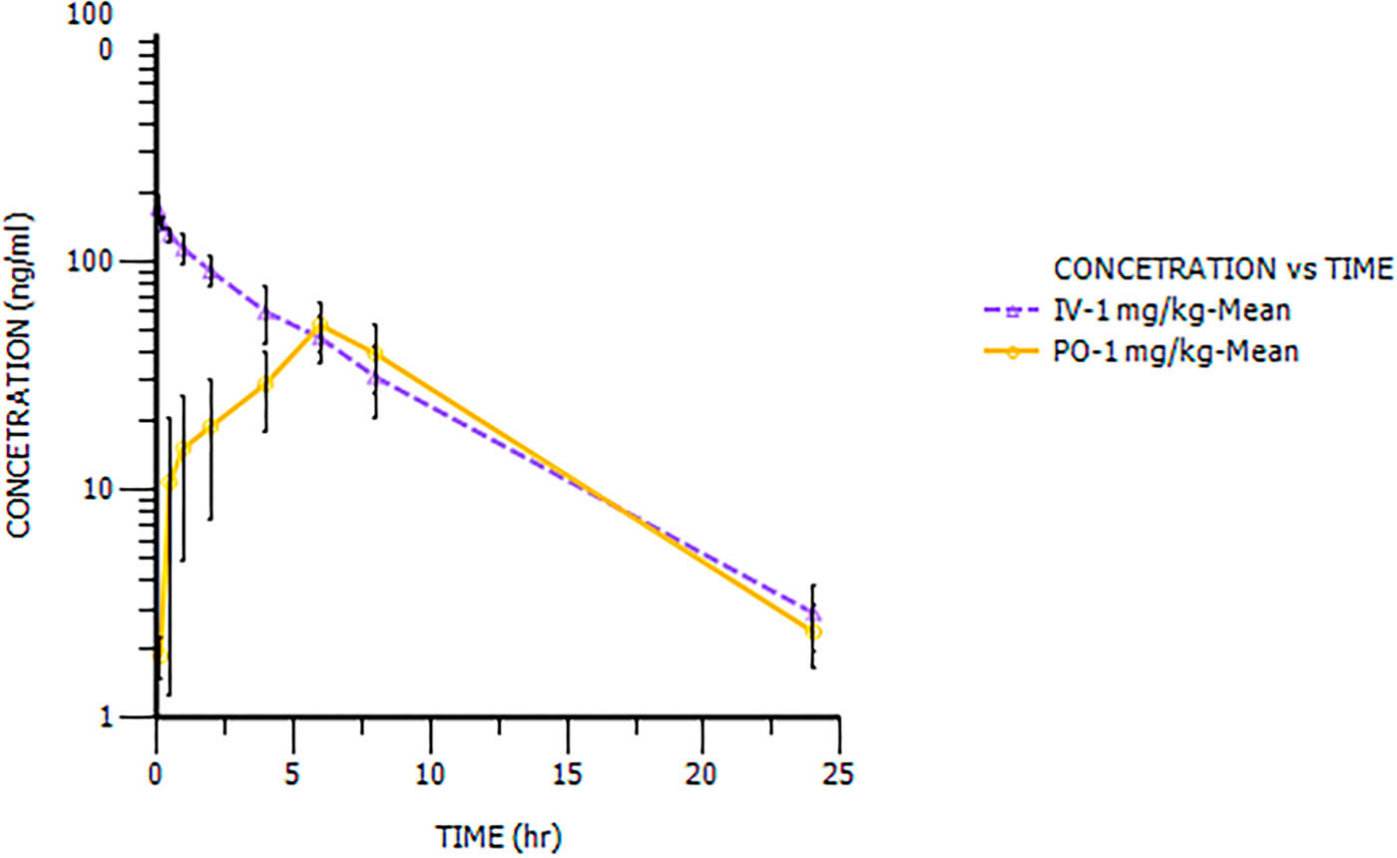
The plasma concentration–time profiles of NHPPC after a single intravenous and oral dose in rats.

**Figure 4.**
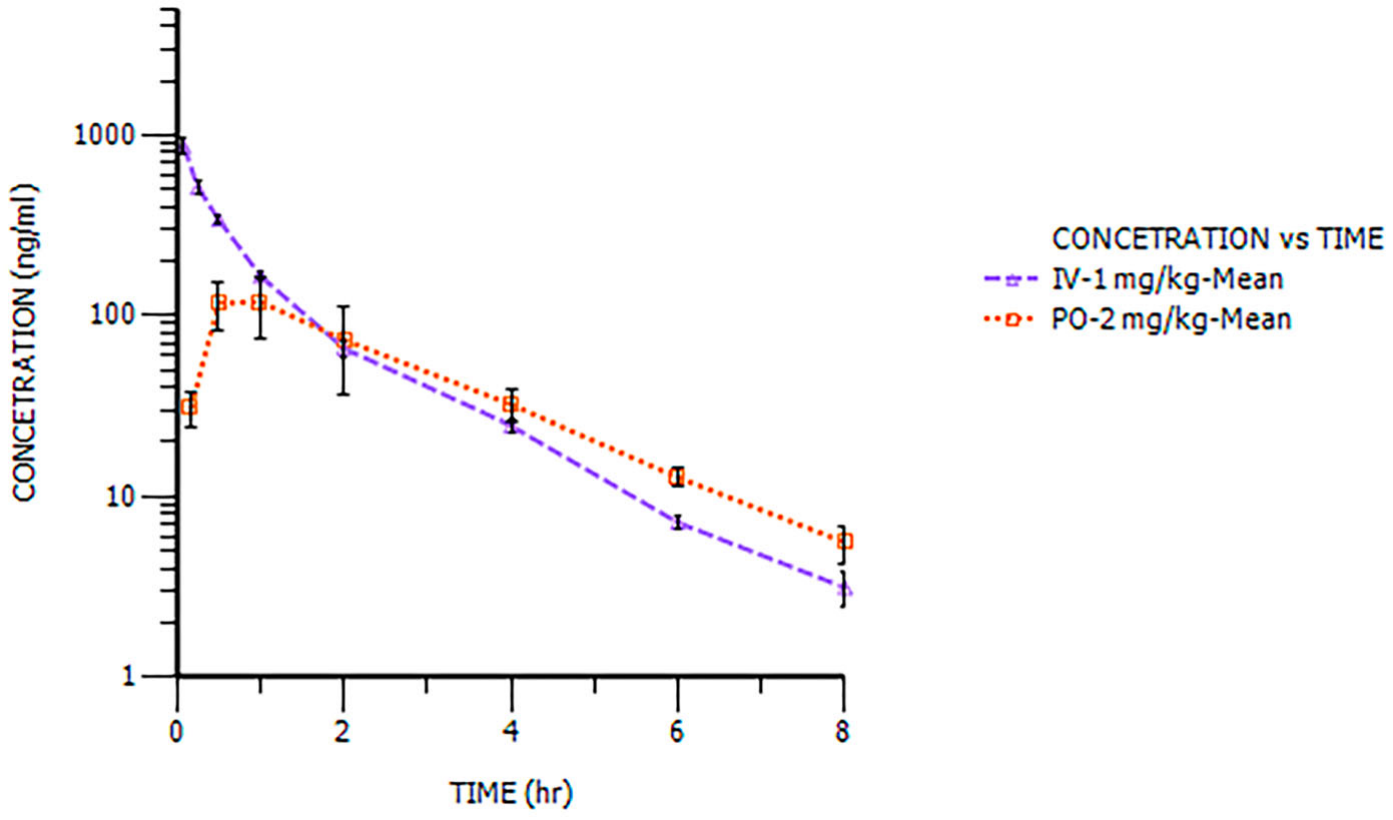
The plasma concentration–time profiles of NHPPC after a single intravenous and oral dose in dogs.

The plasma concentration–time profiles of NHPPC after a single IV and a single PO in rats and dogs were showed in Figures 3 and 4 and the pharmacokinetic parameters were calculated and summarized in Table 4. After IV at 1 mg/kg, the total plasma CL of NHPPC was similar values for both species (1.19 L/h/kg in rats and 1.46 L/h/kg in dogs). The steady state volume of distribution (*V_ss_*) was higher in rats (6.50 L/kg) than in dogs (1.68 L/kg), so the terminal plasma elimination *t*_1/2_ was 4.55 h in rats and 1.33 h in dogs, respectively. After PO, NHPPC was rapidly absorbed with a *T*_max_ between 0.5 and 1.0 h in dogs, while *T*_max_ was 6 h in rats. The *t*_1/2_ values in rats and dogs were 4.02 and 1.57 h for PO. NHPPC absolute bioavailability (BA) in rats and dogs were approximately 34.5% and 53.1%, respectively. The elimination rate constant (K_10_) of NHPPC after PO was consistent with that after IV and dose design were very similar for both species. It indicated that the BA was credible. From Cl_int, in vivo_ and CL_int, in vitro_, the result manifested that there was in vivo and in vitro correlation in rat.

**Table 4.** Pharmacokinetic parameters of NHPPC in plasma after a single intravenous dose and a single oral dose in rats and dogs (each value represents the mean ± SD of three animals).

Species	Rout/Dose (mg/kg)	*K*_10_ (hr^−1^)	*t*_1/2_ (hr)		CL (L/hr/kg)	*V_s_*_s_ (L/kg)	AUC_0–*t*_ (hr*ng/mL)	AUC_0-∞_ (hr*ng/mL)	MRT_0-∞_ (hr)
Rat	IV: 1	0.15 ± 0.00	4.55 ± 0.11		1.19 ± 0.25	6.50 ± 1.00	847 ± 195	866 ± 201	5.50 ± 0.36
Dog	IV: 1	0.52 ± 0.03	1.33 ± 0.06		1.46 ± 0.09	1.68 ± 0.08	679 ± 38.5	685 ± 40.0	1.15 ± 0.04

Species	Rout/Dose (mg/kg)	*K*_10_ (hr^−1^)	*t*_1/2_ (hr)	*T*_max_ (hr)	*C*_max_ (hr*ng/mL)	AUC_0–*t*_ (hr*ng/mL)	AUC_0-∞_ (hr*ng/mL)	MRT_0-∞_ (hr)	BA (%)

Rat	PO: 2	0.17 ± 0.02	4.02 ± 0.39	6.00	52.6 ± 12.9	583 ± 135	593 ± 137	7.80 ± 0.79	34.5 ± 7.91
Dog	PO: 1	0.45 ± 0.06	1.57 ± 0.22	0.50	144 ± 35.3	351 ± 96.1	364 ± 97.4	2.54 ± 0.15	53.1 ± 14.2

## 4. Discussion

The results of PPB indicated that only a very small amount of unbound NHPPC was available in blood for its therapeutic action. The percentage of drug-plasma protein binding was measured in vitro to provide a basis for estimating potential free drug concentrations in vivo. According to the ranking criteria for clearance in different species (Houston 1994; Kerns and Di 2008), NHPPC was high clearance in dog and middle clearance in rat and human. In general industry practice, compounds demonstrate high metabolic instability if the remaining is less than 30%. Based on drug–drug interaction evaluation, NHPPC is expected to have no or low possibility in CYP450-mediated drug–drug interaction in humans. The elimination rate constant (K_10_) of NHPPC after PO was consistent with that after IV and dose design were very similar for both species. It indicated that the BA was credible.

## 5. Conclusions

The science of preclinical drug development is a risk-based exercise that extrapolates nonhuman safety and efficacy information to a potential human outcome. So in the present work, the potential of NHPPC was investigated in preclinical evaluation of drug metabolism and pharmacokinetics. NHPPC was highly bound to plasma proteins. From the in vitro microsomal incubation studies, the result showed high metabolic instability and there is no correlation in vitro and in vivo study and the good correlation appears to have been observed in rats. According to the reported criteria (IC50 > 10 μM categorized as weak inhibition, 1 μM < IC50 < 10 μM categorized as medium inhibition and IC50 < 1 μM categorized as potent inhibition), NHPPC had little inhibition on CYP1A2, CYP2C9, CYP2C19, CYP2D6 and CYP3A4. The results using human primary hepatocytes showed that NHPPC had no induction effect on CYP1A2 or CYP3A4. Based on the data, NHPPC is safe and unlikely to cause any clinically significant herb-drug interactions and thus cause the occurrence of adverse drug reactions in humans when co-adminstered with substrates of the five CYPs. NHPPC was found to be a rapidly absorbed, low clearance and good oral bioavailability compound in dogs. While the result showed that NHPPC has a longer plasma half-life in rats. In addition, this is the first report on plasma protein binding, metabolic stability and pharmacokinetic of NHPPC and provide important leads for studying PDE5 inhibitors.

## References

[CIT0001] Baranczewski P, Stanczak A, Sundberg K, Svensson R, Wallin A, Jansson J, Garberg P, Postlind H. 2006 Introduction to in vitro estimation of metabolic stability and drug interactions of new chemical entities in drug discovery and development. Pharmacol Rep. 58:453–472. 16963792

[CIT0002] Belkoff LH, McCullough A, Goldstein A, Goldstein I, Jones L, Bowden CH, DiDonato K, Trask B, Day WW. 2013 An open-label, long-term evaluation of the safety, efficacy and tolerability of avanafil in male patients with mild to severe erectile dysfunction. Int J Clin Pract. 67:333–341. doi: 10.1111/ijcp.12065 23521325

[CIT0003] Clark DE, Grootenhuis PD. 2002 Progress in computational methods for the prediction of ADMET properties. Curr Opin Drug Discov Dev. 5:382–390. 12058613

[CIT0004] Doggrell SA. 2005 Comparison of clinical trials with sildenafil, vardenafil and tadalafil in erectile dysfunction. Expert Opin Pharmacother. 6:75–84. doi: 10.1517/14656566.6.1.75 15709885

[CIT0005] Houston JB. 1994 Utility of in vitro drug metabolism data in predicting in vivo metabolic clearance. Biochem Pharmacol. 47:1469–1479. doi: 10.1016/0006-2952(94)90520-7 8185657

[CIT0006] Kerns EH, Di L. 2008 Disposition, metabolism, and safety. In: Drug-like Properties: Concepts, structure design and methods. Part 3 Oxford: Elsevier.

[CIT0007] Lave TH, Dupin S, Schmitt C, Chou RC, Jaeck D, Coassolo PH. 1997 Integration of in vitro data into allometric scaling to predict hepatic metabolic clearance in man: application to 10 extensively metabolized drugs. J Pharm Sei. 86:584–590. doi: 10.1021/js960440h 9145383

[CIT0008] Limin M, Johnsen N, Wayne JGH. 2010 Avanafil, a new rapid-onset phosphodiesterase 5 inhibitor for the treatment of erectile dysfunction. Expert Opin Invest Drugs. 19:1427–1437. doi: 10.1517/13543784.2010.518955 20939743

[CIT0009] Selvin E, Burnett AL, Platz EA. 2007 Prevalence and risk factors for erectile dysfunction in the US. Am J Med. 120:151–157. doi: 10.1016/j.amjmed.2006.06.010 17275456

[CIT0010] US FDA (2012-2-1) Guidance for industry: drug interaction studies-study design, data analysis, implications for dosing, and labeling recommendations [EB/OL]. www.fda.gov/downloads/DrugsGuidanceComplianceRegulatoryInformation/Guidances/UCM292362.pdf. 10.1038/sj.clpt.6100054 17259955

[CIT0011] Vande WH, Gifford E. 2003 ADMET in silico modelling: towards prediction paradise? Nat. Rev. Drug Discov. 2:192–204. doi: 10.1038/nrd1032 12612645

[CIT0012] White RE. 2000 High-throughput screening in drug metabolism and pharmacokinetic support of drug discovery. Annu Rev Pharmacol. Toxicol. 40:133–157. doi: 10.1146/annurev.pharmtox.40.1.133 10836130

[CIT0013] Xu R, Nemes C, Jenkins KM, Rourick RA, Kassel DB, Liu CZC. 2002 Application of parallel liquid chromatography/mass spectrometry for high throughput microsomal stability screening of compound libraries. J. Am. Soc. Mass Spectrom. 13:155–165. doi: 10.1016/S1044-0305(01)00342-7 11841071

[CIT0014] Yan Z, Caldwell G W. 2001 Metabolism profiling, and cytochrome P450 inhibition & induction in drug discovery. Curr Top Med Chem. 5:403–425. 10.2174/1568026013395001 11899105

[CIT0015] Yuan JQ, Zhang RJ, Yang ZY, Lee J, Liu YL, Tian JH. 2013 Comparative effectiveness and safety of oral phosphodiesterase type 5 inhibitors for erectile dysfunction: a systematic review and network meta-analysis. Eur Urol. 63:902–912. doi: 10.1016/j.eururo.2013.01.012 23395275

[CIT0016] Zhang D, Zhu M, Humphreys WG. 2007 Drug metabolism in drug design and development. New Jersey: Wiley-InterScience.

[CIT0017] Zhao C, Kim SW, Yang DY, Kim JJ, Park NC, Lee SW, Paick JS, Ahn TY, Moon KH, Chung WS, et al. 2012 Efficacy and safety of avanafil for treating erectile dysfunction: results of a multicentre, randomized, double-blind, placebo-controlled trial. BJU Int. 110:1801–1806. doi: 10.1111/j.1464-410X.2012.11095.x 22448738

